# Serum-level variation in testosterone (steroid hormones) and mood-related symptoms

**DOI:** 10.1192/bjb.2024.128

**Published:** 2025-02

**Authors:** Mostafa Bastawy

**Affiliations:** general adult psychiatry consultant, Commmunity Mental Health Team, Lancashire and South Cumbria NHS Foundation Trust, UK. Email: mostafa.bastawy@lscft.nhs.uk

According to the guidelines of the Endocrine Society, the clinical manifestations of low testosterone in men include both specific signs and symptoms (incomplete sexual development, loss of body hair, small testes, low sperm count) and non-specific signs and symptoms. These non-specific signs are indistinguishable from those of mood and anxiety disorders typically treated in out-patient psychiatric settings.^1^ It is also known that sex steroids influence human behaviour and are associated with conditions including premenstrual dysphoria, psychiatric disorders and depressive symptoms during the postmenopausal period. Another study found that elevated serum testosterone was associated with diagnosis of depression disorder among women with polycystic ovary syndrome.^2^

Several case reports indicate that sex steroid hormones contribute to mood changes. A study in India recruited 103 postpartum women, 62 of whom had been diagnosed with postpartum depression (PPD) according to the Edinburgh PPD scale (score >10). Comparison of the two cohorts (those who developed PPD and those who did not) showed significant elevation of testosterone but not oestradiol or progesterone levels. The researchers used receiver operating characteristic analysis, and found that testosterone levels greater than 42.71 ng/mL at 24–28 h postpartum predicted development of PPD, with 79% sensitivity, 63% specificity, 68% positive predictive value, 74% negative predictive value and area under the curve of 0.708.^3^ In men, studies have shown the contrary, with low testosterone levels associated with signs and symptoms of major depression. There is significant inverse correlation of bioavailable testosterone (but not total testosterone) levels and depression scores in elderly men, independent of age and weight.

In another study by Schmidt et al, the depressive symptoms associated with a hypogonadal state were reversed by testosterone replacement.^4^ These symptoms included reduced libido and reduced feelings of well-being. Kratzik et al found an elevated prevalence of depression in both men with increased testosterone level and those with low levels. However, this effect was only detectable in underweight and obese men, not in men of ‘normal’ weight, in whom a linear decrease in the risk of depression with increasing testosterone level was noted.^5^

My conclusion is in agreement with a literature review conducted in 2018, which suggested a lack of proper epidemiological research on low testosterone in patients presenting with psychiatric complaints. This is despite the fact that there has been shown to be a significant overlap in clinical symptoms. Identification of low testosterone could have a significant impact on treatment through urologic referral for testosterone repletion or the use of treatments that spare the gonadal axis.^6^
